# A Possible Preventive of Rabies

**Published:** 1881-12

**Authors:** 


### A POSSIBLE PREVENTIVE OF RABIES.

In the *New York Medical Journal and Obstetrical Review* for November, 1881, Dr. L. L. Dorr, of San Francisco, discusses the etiology of hydrophobia, with special reference to climatic influence. The Pacific coast of America, he remarks, presents some peculiar features as regards this disease: from Behring's Strait to Cape Horn, he states, not a well-authenticated case of rabies was ever known. In support of this statement he adduces testimony to which great weight must be allowed. He therefore dissents from the common opinion that climate has no influence over the disease. A test of the power of the climate of the American Pacific coast to prevent the development of rabies can easily be made, he suggests, with strong probability of proving a blessing to hundreds of human beings who have no other hope. The period of incubation is so long in many cases that several dogs known to be badly bitten by a dog known to be suffering with rabies could be sent there under guard and kept behind prison-bars and under observation until the extreme limit was passed. Or a more practicable plan might be to have a number of the many persons who are annually bitten by rabid dogs in the Eastern States and Europe go to that coast, and remain there until the extreme limit of the possibilities was passed, selecting any place from Cape Horn to Behring's Strait—preferably, however, California. where they have varied climate and all kinds of dogs, and are in close communication with all parts of the world. The patient having a knowledge of these facts, and the support of hope of escape, it might be a power to prevent an attack ; and, if he were attacked, the disease might be so modified by the climate as to render it susceptible of cure.

